# Phenotypic selection under two contrasting environments in wild sunflower and its crop–wild hybrid

**DOI:** 10.1111/eva.12828

**Published:** 2019-07-01

**Authors:** Alejandro Presotto, Fernando Hernández, Kristin L. Mercer

**Affiliations:** ^1^ Centro de Recursos Naturales Renovables de la Zona Semiárida (CERZOS), Departamento de Agronomía Universidad Nacional del Sur (UNS)‐ CONICET Bahía Blanca Buenos Aires Argentina; ^2^ Department of Horticulture and Crop Science Ohio State University Columbus OH

**Keywords:** competition, crop–wild hybrids, genotype‐by‐environment interactions, introgression, linear, morphological traits, quadratic, selection

## Abstract

Hybridization is a common phenomenon in plants and can lead to the introgression of alleles from one population into another, generate new hybrid lineages, or cause species extinction. The environmental conditions and the genetic background of the participating populations may influence these outcomes since they can affect the fitness of hybrids, thereby increasing or decreasing the chances of introgression. Thus, it is important to understand the context‐dependent prospects for introgression of alleles into diverse populations and under multiple ecological environments. Crop–wild hybridization presents an opportunity to explore these dynamics in agroecosystems. To this end, we used diverse wild and hybrid sunflowers from across the northern United States as a basis for evaluating variation in morphological traits and assessing context‐dependent selection. These crop–wild hybrids and their wild counterparts were grown under agricultural conditions in the field with and without wheat competition. Interactions between origin and cross type affected expression of early functional traits, while interactions between competition and cross type acted on reproductive traits. A smattering of early and reproductive traits was affected by interactions between cross type and competition that varied by origin (i.e., 3‐way interactions). Seven functional traits, especially number of branches and tertiary head diameter, underwent net and direct directional selection, while six out of these seven traits appear to also be experiencing nonlinear selection dynamics. In general, wild‐like traits were favored under both sets of conditions, while, under wheat competition, some crop‐like traits related to fast growth and primary head diameter became important. These data reaffirm the hypothesis that stressful conditions establish a scenario more suitable for crop introgression and clarify that nonlinear selection dynamics may play a role in this process.

## 1 INTRODUCTION

Hybridization is a frequent and important component of plant evolution and speciation (Rieseberg, Ellstrand, & Arnold, 1993). To hybridize, populations must occupy the same area, overlap in flowering time, and share pollinators (or together be exposed to other pollen vectors). After that, the process of introgression depends on fitness of hybrids sufficient for them to backcross with one of their parents, cross with another hybrid, or self‐pollinate. If novel alleles reduce hybrid fitness, these alleles may not prevail or introgress (Jarvis & Hodgkin, 1999). While these requirements appear stringent, there are many reports of introgression of novel alleles in hybrid zones (Arnold, 1992; Barton & Hewitt, 1985; Martin, Bouck, & Arnold, 2006; Pardo‐Diaz et al., 2012; Whitney, Randell, & Rieseberg, 2006). While this has been documented in natural systems, it is perhaps becoming even better studied in the context of hybridization between crops and neighboring populations of wild relatives: for example, in radish (Snow et al., 2010), rice (Dong et al., 2017), oilseed rape (Warwick, Légère, Simard, & James, 2008), lettuce (Uwimana et al., 2012), sunflower (Whitton, Wolf, Arias, Snow, & Rieseberg, 1997), carrot (Shim & Jørgensen, 2000), wheat (Kwit, Moon, Warwick, & Stewart, 2011), and maize (Trtikova et al., 2017). This crop–wild introgression can lead to the formation of new hybrid lineages (Abbott et al., 2013; Payseur & Rieseberg, 2016) or may cause extinction through the replacement of one or both parents (genetic swamping) or the loss of genetic diversity (Ellstrand, Prentice, & Hancock, 1999; Gómez et al., 2015; Todesco et al., 2016). However, we still need to better understand the process of adaptive evolution that can accompany introgression.

All crops have been domesticated from wild progenitor species and have undergone evolutionary changes that increased their fitness under cultivation, while concurrently making them dependent on humans for survival (Meyer & Purugganan, 2013; Purugganan & Fuller, 2009). Many traits are commonly associated with domestication, including lack of seed/fruit dormancy, seed/fruit retention, large seed/fruit size, reduced or no branching, reduced plant height, and synchronized flowering and seed maturation (Doebley, Gaut, & Smith, 2006; Hernández, Lindström, Parodi, Poverene, & Presotto, 2017; Meyer & Purugganan, 2013). Most of these traits are considered “maladaptive” in noncultivated environments, and this could be one of the reasons for the low fitness seen in most crop–wild hybrids (Mercer, Wyse, & Shaw, 2006; Snow, Moran‐Palma, Rieseberg, Wszelaki, & Seiler, 1998; Spencer & Snow, 2001; Zhi, Lu, Wang, & Jia, 2004). However, some crop‐like traits, such as large leaves, numerous inflorescences and seeds, rapid growth, and self‐compatibility, can be favorable in a range of environments (Arnaud, Fénart, Cordellier, & Cuguen, 2010; Baack, Sapir, Chapman, Burke, & Rieseberg, 2008; Dechaine et al., 2009; Mercer, Andow, Wyse, & Shaw, 2007), while others may increase susceptibility to herbivory (Alexander, Cummings, Kahn, & Snow, 2001; Dechaine et al., 2009; Presotto, Pandolfo, Poverene, & Cantamutto, 2016). Thus, introgression of crop‐like traits into wild populations may be beneficial or detrimental depending on the trait and the species.

Environmental factors can strongly affect fitness in crop–wild hybrids, as well as their fitness relative to their wild counterparts, thereby increasing or decreasing the chances of introgression of crop alleles into wild populations. Due to this context dependence, environments that exert positive selection on crop‐like traits could increase the chances of introgression of the underlying alleles (e.g., under crop competition, herbicide selection) (Campbell & Snow, 2007; Hartman et al., 2013; Hovick, Campbell, Snow, & Whitney, 2012; Londo, Bollman, Sagers, Lee, & Watrud, 2011; Mercer et al., 2007; Owart, Corbi, Burke, & Dechaine, 2014; Uwimana et al., 2012). In addition, the fitness effects of the same crop alleles into different wild genetic backgrounds may affect the chances of their introgression since various genetic combinations may display divergent fitness (Mercer et al., 2007, 2006; Presotto, Ureta, Cantamutto, & Poverene, 2012; Snow et al., 1998; Xia et al., 2016). For example, Mercer et al. (2006) found that wild sunflower fecundity declined more severely under stressful conditions than did that of their hybrid counterparts. Specifically, without added stressors, fitness of hybrids relative to wilds (i.e., fitness of hybrids as a proportion of the fitness of wilds) ranged from 0.1 to 0.5 across genetic backgrounds, while under stressful conditions, relative hybrid fitness ranged more broadly between 0.3 and 1.1, likely due to faster early growth in hybrids, a crop‐like trait (Mercer et al., 2007). Thus, environmental and genetic factors contribute to the context dependence of the chances of introgression of crop alleles into wild populations. Insight into the likelihood of various introgression scenarios depends on improved understanding of the selection dynamics.

Selection acts on traits, and those correlated with them, based on their genetically based impact on fitness, thereby affecting the phenotypic distribution of a trait in a population over the generations (Brodie, Moore, & Janzen, 1995). Phenotypic selection analysis is a useful method for estimating the strength of direct and indirect selection on quantitative traits as it can indicate the degree to which fitness in a particular habitat or environment depends on individual traits or suites of traits (Kingsolver et al., 2001; Lande & Arnold, 1983; Mitchell‐Olds & Shaw, 1987). This methodology has been useful to advance in knowledge of the selection dynamics in wild and crop–wild hybrids under different scenarios (Baack et al., 2008; Campbell, Snow, & Sweeney, 2009; Dechaine et al., 2009; Emel, Franks, & Spigler, 2017; Exposito‐Alonso, Brennan, Alonso‐Blanco, & Picó, 2018; Parachnowitsch & Kessler, 2010; Wu & Li, 2017). For example, Wu & Li, 2017, demonstrated that variation in pollinator assemblages in *Primula secundiflora* not only results in variation in the strength of selection, but also in the direction of selection (e.g., early or late flowering), indicating the importance of pollinator‐mediated selection on floral evolution. In wild sunflower, crop hybridization has also been shown to affect selection dynamics, suggesting the possibility of introgression of crop alleles underlying larger plant and inflorescence size (e.g., larger leaves) and/or fast growth, as well as earlier emergence, especially via particular cross types (Baack et al., 2008; Dechaine et al., 2009; Kost, Alexander, Jason Emry, & Mercer, 2015). However, we do not adequately understand the context dependence of that selection.

Here, using the fecundity data analyzed in Mercer et al. (2006), Mercer et al. (2007) and associated, largely unanalyzed trait data on wild and crop–wild hybrid sunflowers from a diverse set of populations, we study the phenotypic variation of functional traits, as well as selection operating on them, under two contrasting environments. Specifically, we:

1. Evaluate the effect of genetic background (i.e., wild population origin), competitive environment, cross type (i.e., wild vs. F1 crop–wild hybrid), and their interactions on different functional plant traits.
2. Determine direct and indirect selection acting on this range of traits and how selection differs in wild and hybrid cross types with and without competition.

By understanding more about variation in, and selection operating on, functional traits in distinct environmental and genetic contexts, we can better understand what differential selection might mean for evolutionary potential.

## 2 MATERIALS AND METHODS

### 2.1 Study system

Sunflower is an excellent system to better understand phenotypic variation across recipient wild populations and how selection on crop‐like traits may proceed after hybridization. Sunflower (*Helianthus annuus*), one of the main oilseed crops globally, was domesticated more than 4,500 years BP (Purugganan & Fuller, 2009) from wild *H. annuus* (hereafter, wild sunflower) populations in eastern North America (Blackman et al., 2011). In the post‐Columbian era, the domesticated sunflower was introduced to Europe, improved in Russia, and then distributed worldwide; 75% of the 40 million tons produced currently is grown in Ukraine, Russia, European Union, and Argentina (www.fas.usda.gov). Otherwise, sunflower production in the United States is relatively small (~1.2 million tons) and it is concentrated in the Northern Great Plains (North Dakota, South Dakota, and Minnesota states) where hybridization with wild sunflower is common (Arias & Rieseberg, 1994; Burke, Gardner, & Rieseberg, 2002). Wild sunflower has been transported from North America into several regions of the world, such as South America, Europe, and Australia (Dry & Burdon, 1986; Lentz, Bye, & Sánchez‐Cordero, 2008; Muller et al., 2009; Poverene et al., 2002), where it now coexists with its cultivated congener, increasing the possibility of gene flow between crop and wild populations globally (Ureta, Carrera, Cantamutto, & Poverene, 2008).

### 2.2 Plant material and experimental design

The wild sunflower plant populations were sourced from USDA accessions originating in the northern Great Plains of the United States: Idaho (ID), Iowa (IO), Minnesota (MN), Montana (MT), North Dakota (ND), South Dakota (SD), Washington (WA), Wyoming (WYI), and Wyoming (WYII) [see Mercer et al. (2006) for more information on these populations and crosses]. In 2002 in St. Paul, MN, we reproduced each of these wild populations by performing hand‐pollinations between randomly paired plants within the population and generated F1 crop–wild hybrids from each by pollinating randomly chosen wild plants with crop pollen from one of three inbred lines: HA 89, HA 425, and SU‐B (from the USDA Breeding Program, Fargo, ND). Each cross type (wild or F1 hybrid, hereafter hybrid) from each population was represented by 15–20 bulked maternal families.

Our 2003 field experiment was designed as a split plot with eight replications (one of which was not analyzed, see below) on the St. Paul Experiment Station of the University of Minnesota, as described in Mercer et al. (2006). To the main plots within each block, four treatments were randomly assigned: (a) wheat competition; (b) sulfonylurea (SU) herbicide application (field rate); (c) SU herbicide application (three times the field rate); and (d) a no competition, no herbicide control. Here, we discuss only results from the control and competition treatments. Subplots were randomly assigned in each main plot to the 36 different cross types, that is, progeny from the wild–wild or the three kinds of crop–wild crosses. Each subplot was composed of four individuals representing a given cross type.

Germinated sunflower seeds were planted 1.8 m apart in rows, with four planting positions per subplot, 10 subplots per row, and four rows per main plot. Prior to sunflower planting, the competition treatment was planted as 10, 15‐cm‐spaced rows of the wheat (*Triticum aestivum* L.) cultivar, Alsen, surrounding each row of sunflower planting positions. The wheat was planted at double the normal seeding rate (at 202 kg/ha) due to wheat's sensitivity to the herbicide, trifluralin, applied prior to sowing. For the control treatment, the planting pattern was the same as the competition treatment, but without wheat. During the experiment (June), a rainfall event of 15 cm damaged, uprooted, or buried many plants in replication two. Thus, replication two was removed from the analysis (for more details see Mercer et al., 2006).

### 2.3 Morphological measurements

Twelve traits were evaluated in the present work: early and intermediate plant height; early and intermediate leaf length; days to flowering; number of branches; primary, secondary, and tertiary head diameter; normal and deformed head number; and overall appearance. Early and intermediate traits were evaluated on each plant two and five weeks after planting, respectively. Days to flowering was noted at first anthesis, and the diameters of the primary, one secondary, and one tertiary head per plant were measured at the widest point across each head's fully pollinated florets. On each plant, the number of branches and numbers of normal and deformed heads were counted at harvest. We used appearance, a categorical trait, to holistically evaluate how wild‐, crop‐, or hybrid‐like plants appeared as a whole, including impressions of head size, branch numbers, branching patterns, head shape, and stem appearance (Table S1). The fitness of the plants (seeds per plant and survival to reproduction), per se, was analyzed in previous publications (Mercer et al., 2007, 2006). However, here we used an estimation of seeds per reproductive plant [based on number of heads x seeds per head (estimated from head diameters); see Mercer et al. (2006) for thorough description] as a dependent variable in our selection analyses.

### 2.4 Statistical analysis

All data were analyzed using generalized linear mixed models (GLMMs) with restricted maximum likelihood in PROC GLIMMIX (SAS, University edition). Our first set of analyses discerned the effects of experimental factors on trait values. Competition, origin, cross type, and their two‐ and three‐way interactions were considered as fixed effects, while block, block by competition, and block by origin by cross type by competition were considered as random—the latter two acting as error terms for main plot and subplot factors, respectively. All wild and hybrid plants were used in these analyses, and the three hybrid types were analyzed jointly. To satisfy assumptions regarding normality of residuals, we used the square root transformation when needed.

In our second set of analyses, we performed phenotypic selection analysis under two environments (control and wheat competition) by employing regressions to estimate selection coefficients on the relationship between fecundity and individual (*s*: selection differentials) or multiple (*β*: selection gradients) traits using a GLMM analysis. Selection differentials (*s*) represent the combination of direct and indirect selection, while selection gradients (*β*) represent direct selection. To avoid collinearity in highly correlated traits, for traits with *r* > 0.5 in all four combinations of cross type and competition, one trait of the pair was retained (Lande & Arnold, 1983). We standardized all variables (including fecundity) to a mean of 0 and a standard deviation (*SD*) of 1 to allow direct comparison of the strength and direction of selection among traits (Wolfe & Tonsor, 2014).

The basic model for univariate and multivariate linear selection analyses built on the one mentioned above. To estimate standardized selection differentials (*s'*) individually for each trait, each trait and its interactions with cross type, competition, and cross type and competition were included in separate models (i.e., different models for each trait). By contrast, to estimate standardized selection gradients (*β')*, all traits and their interactions with cross type, with competition, and with cross type and treatment were included in a single model that allowed for covariances among traits to be analyzed. Due to significant interactions, we produced selection differentials and gradients by competition, by cross type, and by the combination of competition and cross type.

In our third set of analyses, we estimated standardized nonlinear selection differentials (*C*′) and gradients (*γ*′) with quadratic terms of single traits analyzed singly (*C*′) or of single traits analyzed jointly (*γ*′) included in nonlinear regression models. While linear selection coefficients measure directional selection and indicate whether selection favors larger or smaller trait values, quadratic coefficients measure curvature in the trait–fitness relationship (Geber & Griffen, 2003; Lande & Arnold, 1983). Analysis of nonlinear selection can help us identify potential stabilizing (negative quadratic coefficients) or disruptive (positive quadratic coefficients) selection (Parachnowitsch & Kessler, 2010; Wu & Li, 2017); however, their interpretation can be complex (Shaw & Geyer, 2010). In order to obtain appropriate coefficients from the quadratic regression model, we doubled the quadratic selection coefficients (Stinchcombe, Agrawal, Hohenlohe, Arnold, & Blows, 2008). So, *C*′ = 2q and *γ*′ = 2q, q being the quadratic term from simple and multiple nonlinear regression, respectively. As in the linear selection analyses, we obtained *C*′ and *γ*′ from the overall data (including the interactions of each trait with cross type, competition, and cross type by competition) (Table 3).

## 3 RESULTS

### 3.1 Effects of competition, origin, cross type, and their interactions

**Figure 1:**
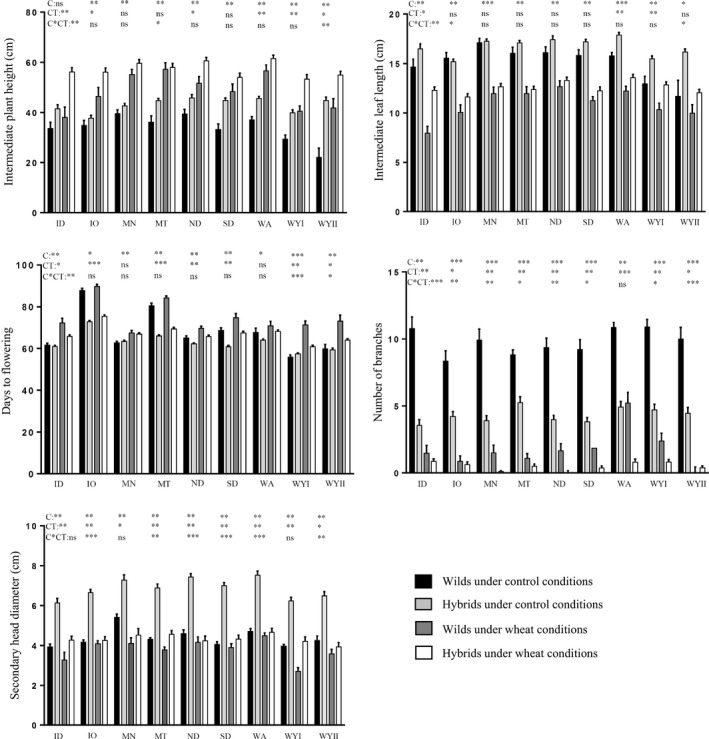
Intermediate plant height and leaf length, days to flowering, number of branches, and secondary (means ± *SE*) head diameter for wilds and hybrids under control and wheat competition from each maternal origin. ID, Idaho; IO, Iowa; MN, Minnesota; MT, Montana; ND, North Dakota; SD, South Dakota; WA, Washington; WYI and WYII, Wyoming. Competition (C), cross type (CT), and C*CT interaction effects are shown for each maternal origin on the upper part of each chart

All the traits showed significant main effects of competition (except for number of deformed heads), origin, and cross type (except for number of tertiary heads). Early growth traits such as early leaf length (ELL), early plant height (EPH), intermediate leaf length (ILL), and intermediate plant height (IPH) showed significant origin‐by‐cross type interactions, meaning that the effect of hybridization depended on their origin. For reproductive traits, such as number of branches (BRN), primary head diameter (PHD), secondary head diameter (SHD), and tertiary head diameter (THD), the competition‐by‐cross type interaction became important, indicating that the effect of cross type depended on the competitive environment. In days to flowering (DFL), number of branches (BRN), and normal head number (NHN), all the interactions were significant, while in appearance (APR) none were (Table 1). In fact, five of the 12 traits showed significant three‐way interactions, indicating that the way that the effects of hybridization differed by competitive environment depended on the origin, so re‐analyses were performed for each population origin separately (Figure 1). In this section, we only show the responses of some of the traits affected by significant interactions (two‐way and three‐way) due to similar patterns between them. Specifically, we highlight data from intermediate plant height, intermediate leaf length, number of branches, secondary head diameter, and days to flowering (Figure 1), but not from their correlated traits (early plant height, early leaf length, and normal head number), which showed similar responses.

**Table 1:** Analysis of variance of twelve traits evaluated in nine wild populations and their hybrids with cultivated sunflower under two environments

**Source**	Num *df*	Den *df*	ELL	EPH	ILL	IPH	DFL	BRN	PHD	SHD	THD	NHN	DHN	APR
			*F‐*value	*F‐*value	*F‐*value	*F‐*value	*F‐*value	*F‐*value	*F‐*value	*F‐*value	*F‐*value	*F‐*value	*F‐*value	*F‐*value
Competition (C)	1	6	20.77**	82.20***	134.80***	130.90***	155.11***	285.03***	33.78**	265.56***	69.04**	406.81***	3.50 ns	8.16*
Origin (O)	8	202	8.10***	17.44***	9.85***	9.94***	137.77***	7.38***	12.30***	7.69***	5.49***	9.90***	2.05*	2.65*
Cross type (CT)	1	202	5.91*	43.30**	58.75***	105.92***	288.97***	339.98***	557.18***	196.84***	0.01 ns	699.43***	279.30***	465.36***
C*O	8	202	0.81 ns	1.85 ns	2.37*	0.97 ns	6.43***	1.85 ns	1.84 ns	0.94 ns	0.53 ns	4.25***	0.64 ns	0.84 ns
C*CT	1	202	0.77 ns	3.37 ns	0.09 ns	0.64 ns	16.12***	61.29***	5.13*	76.35***	7.68**	152.19***	14.23**	0.19 ns
O*CT	8	202	5.47***	5.40***	4.07**	4.13**	22.14***	3.10**	0.94 ns	1.07 ns	1.86 ns	3.86**	0.78 ns	1.59 ns
C*O*CT	8	202	1.78 ns	2.38*	1.87 ns	2.26*	4.94***	2.59*	1.31 ns	1.75 ns	1.09 ns	2.02*	1.06 ns	0.59 ns

Abbreviations: APR, appearance; BRN, number of branches; DFL, days to flowering; DHN, deformed head number; ELL, early leaf length; EPH, early plant height; ILL, intermediate leaf length; IPH, intermediate plant height; NHN, normal head number; PHD, primary head diameter; SHD, secondary head diameter; THD, tertiary head diameter.

ns, nonsignificant.

* *p* < 0.05.

** *p* < 0.01.

*** *p* < 0.001.

Competition generally reduced leaf length, branching, head diameter, and number of heads, but it increased plant height and days to flowering (Table 1, Figure 1). Similarly, hybrids were generally taller and had longer leaves, fewer branches, and larger heads than wilds and they flowered at a similar time to, or earlier than, wilds (Figure 1). However, for most traits, we found significant interactions of competition with cross type and these varied with origin. For example, in two maternal origins (WYII and MT), the intermediate height of wild plants with competition increased relatively more than that of hybrid plants, while for plants from ID, this relationship was inverted with hybrid plants having a larger response in the face of competition. Similarly, intermediate leaf length declined more with competition in wild ID and IO plants than in their hybrid counterparts; by contrast, WYII hybrids were more affected than their wild counterparts when experiencing competition (Figure 1).

Branching was strongly reduced with competition, especially in wilds, except in the WA population for whom the reduction was similar for both cross types (Figure 1). In plants from most origins, under competition wilds still had more heads than hybrids; however, in IO and WYII populations, wild branching was so reduced under wheat competition that the number of branches was similar in both cross types (Figure 1). Days to flowering was delayed by competition in general, but in ID, WYI, and WYII populations wild plants were more severely delayed in flowering than hybrids (Figure 1). Secondary head diameter was reduced by competition, but hybrids with IO, MT, ND, SD, WA, and WYII populations were more greatly reduced in head diameter than their corresponding wilds (Figure 1).

### 3.2 Phenotypic selection analysis

To avoid collinearity due to highly correlated traits, we only retained seven of the 12 traits for selection analysis (Table 2). We detected linear selection acting on our group of study traits. When traits were analyzed individually using the full model, selection differentials (*s'*) on all traits were significantly different from zero (Table S2) indicating a significant combination of direct and indirect selection on the traits. BHN, as well as THD, experienced the strongest selection, and PHD was the only trait negatively selected upon (Table S2). For most traits, the wild cross type experienced greater selection than the hybrid cross type. By contrast, changes in selection due to competition varied by trait; some traits experienced greater selection with competition (IPH, ILL, SHD), others experienced greater selection under control conditions (BRN, THD), and still others showed opposite direction of selection under the two environments (PHD) (Table S2). However, for most traits (except ILL and DTF), the difference in selection acting on a trait in the two cross types depended on the competition treatment (i.e., the three‐way interaction between trait, competition, and cross type was significant; Table S2). Thus, we emphasize results from subanalyses by the combination of competition and cross type here.

**Table 2:** Selection coefficients for seven sunflower traits calculated by competition (control vs. wheat) treatment, by cross type (wild vs. hybrid), and by the combination of competition and cross type

Trait	By competition					By cross type					By competition and cross type								
	Sig int	Control		Wheat		Sig int	Wild		Hybrid		Sig int	Wild				Hybrid			
												Control		Wheat		Control		Wheat	
		*s*′ ± *SE*		*s*′ ± *SE*			*s*′ ± *SE*		*s*′ ± *SE*			*s*′ ± *SE*		*s*′ ± *SE*		*s*′ ± *SE*		*s*′ ± *SE*	
IPH	***	**0.12**	*0.03*	**0.20**	*0.01*	***	**0.23**	*0.04*	**0.08**	*0.02*	***	**0.27**	*0.08*	**0.24**	*0.03*	0.03	*0.03*	**0.14**	*0.01*
ILL	***	**0.16**	*0.03*	**0.18**	*0.01*	***	**0.25**	*0.03*	**0.09**	*0.01*	ns	**0.24**	*0.06*	**0.25**	*0.02*	0.02	*0.03*	**0.16**	*0.01*
DFL	**	0.05	*0.04*	0.00	*0.01*	ns	0.01	*0.03*	0.00	*0.02*	ns	0.04	*0.07*	0.00	*0.04*	0.02	*0.04*	0.05	*0.02*
BRN	***	**0.57**	*0.04*	**0.33**	*0.01*	***	**0.52**	*0.05*	**0.44**	*0.02*	***	**0.53**	*0.11*	**0.37**	*0.04*	**0.55**	*0.02*	**0.31**	*0.02*
PHD	***	**−0.20**	*0.06*	**0.21**	*0.02*	*	**0.08**	*0.06*	−0.03	*0.01*	***	−0.14	*0.13*	**0.35**	*0.05*	**−0.17**	*0.03*	**0.10**	*0.01*
SHD	***	**0.28**	*0.06*	**0.30**	*0.01*	ns	**0.50**	*0.07*	**0.12**	*0.01*	***	**0.64**	*0.13*	**0.46**	*0.04*	**0.09**	*0.03*	**0.16**	*0.01*
THD	***	**0.63**	*0.04*	**0.28**	*0.02*	***	**0.68**	*0.05*	**0.29**	*0.01*	**	**0.98**	*0.08*	**0.40**	*0.03*	**0.41**	*0.02*	**0.16**	*0.01*
		*β′ ± SE*		*β′ ± SE*			*β′ ± SE*		*β′ ± SE*			*β′ ± SE*		*β′ ± SE*		*β′ ± SE*		*β′ ± SE*	
IPH	*	0.00	*0.03*	0.03	*0.02*	**	0.04	*0.04*	0.00	*0.02*	***	0.10	*0.08*	0.04	*0.04*	−0.03	*0.03*	0.01	*0.01*
ILL	ns	0.02	*0.03*	**0.08**	*0.02*	ns	0.03	*0.04*	0.03	*0.03*	*	0.05	*0.08*	**0.12**	*0.04*	−0.01	*0.03*	**0.04**	*0.02*
DFL	ns	**0.08**	*0.03*	**0.09**	*0.02*	ns	0.06	*0.04*	**0.06**	*0.02*	ns	0.06	*0.06*	**0.12**	*0.03*	0.05	*0.03*	**0.07**	*0.02*
BRN	***	**0.49**	*0.04*	**0.16**	*0.02*	**	**0.38**	*0.05*	**0.33**	*0.22*	**	**0.40**	*0.10*	**0.16**	*0.03*	**0.45**	*0.03*	**0.17**	*0.02*
PHD	***	**−0.21**	*0.04*	0.00	*0.02*	***	**−0.17**	*0.06*	0.02	*0.02*	***	**−0.34**	*0.11*	0,00	*0.04*	−0.01	*0.02*	0.03	*0.01*
SHD	**	−0.04	*0.04*	**0.13**	*0.02*	ns	0.08	*0.08*	0.03	*0.02*	ns	−0.01	*0.02*	**0.17**	*0.06*	−0.04	*0.02*	**0.10**	*0.02*
THD	***	**0.63**	*0.04*	**0.20**	*0.02*	***	**0.62**	*0.06*	**0.23**	*0.01*	***	**0.97**	*0.11*	**0.26**	*0.04*	**0.34**	*0.02*	**0.12**	*0.01*

Note

Standardized selection differentials and selection gradients with their standard errors (*s' ± SE* and *β' ± SE,* respectively) are shown. The columns on the left of each section indicate the significance of the relevant interaction from Table S2 (e.g., the stars to the left of the “By competition” section refer to significant interactions between the trait of interest and competition).

Numbers in bold indicate significant coefficients (*p* < 0.05).

Abbreviations: BRN, number of branches; DFL, days to flowering; ILL, intermediate leaf length; IPH, intermediate plant height; PHD, primary head diameter; SHD, secondary head diameter; THD, tertiary head diameter.

SE values are shown in italic.

* *p* < 0.05.

** *p* < 0.01.

*** *p* < 0.001.

Only a few traits (BRN, SHD, and THD) were subject to significant positive selection (*s'*) in all combinations of environments and cross types (Table 2). For BRN, positive selection was greater for both wild and hybrid cross types under control conditions, although the difference may have been greater for hybrids (Table 2). For SHD, wilds underwent greater selection than hybrids in both competitive environments. THD experienced positive selection that was greater for wild than hybrid cross types and greater for control than competitive environments, such that the values followed this pattern: (control/wild > wheat/wild = control/hybrid > wheat/hybrid). PHD was positively selected for under wheat and negatively selected for under control conditions (nonsignificant for wilds), and the effect of competition on selection appears to be greater for wilds than hybrids. Wilds underwent greater selection on IPH than hybrids, and, for hybrids, selection was greater on this trait with competition.

With regard to selection gradients (*β'*) discerned from models including all traits and their interactions with cross type and competition (Table S2), selection on DTF, BRN, PHD, and THD was significantly different from zero, consistent with direct selection on these traits. Direct selection on BRN and THD was strongest, and PHD was once again the only trait undergoing negative selection (Table S2). In addition, selection on all the traits, except DFL and SHD, varied with the combination of competition and cross type. Positive selection on DFL appeared to be consistent, while selection on SHD differed with competition (Table S2).

Selection gradients (*β'*) varied between wild and hybrid cross types, with direct selection being generally stronger in the wilds, and were affected by competition, with some traits subject to direct selection in one environment but not the other (Table 2). An instance of the latter is SHD; it appeared to be under significant positive direct selection with competition, but nonsignificant negative direct selection without (Table 2). However, for most of these traits, significant three‐way interactions among trait, cross type, and competition indicate even greater complexity; they mean that the effect of competition on the level and/or direction of direct selection depends on cross type. When all traits were analyzed by the combination of competition and cross type, BRN and THD were directly selected for in all cases; however, the magnitude of selection varied with the strongest selection in the control conditions for both cross types and the selection being especially strong for THD in control wilds (Table 2). PHD was directly negatively selected on only in wild plants under control conditions (Table 2). ILL, DFL, and SHD were positively selected under competition with wheat for both cross types (and there was no selection without competition), but the strength of selection with competition for wilds appears to have been slightly greater than for hybrids (Table 2).

We found suggestive evidence for quadratic selection in our study traits, with corresponding coefficients *(C*′) being significantly different from zero for all traits, except for BRN (Table S3). We observed negative *C*′ values for IPH (−0.09 ± 0.01), PHD (−0.13 ± 0.03), and SHD (−0.14 ± 0.06), and positive *C*′ values for ILL (0.08 ± 0.01), DTF (0.08 ± 0.02), and THD (0.21 ± 0.04), suggesting possible stabilizing and disruptive selection, respectively. However, the environment also affected the nature of selection here. Under control conditions, IPH, PHD, and SHD showed negative *C*′ values, which were larger in both PHD and SHD (Table 3). By contrast, under competitive conditions, four of the six traits had positive *C*′ values (Table 3). Interestingly, the nonlinear selection experienced by three traits, IPH, DFL, and SHD, reverted from positive to negative values depending on the environment (Table 3). Cross type alone did not affect selection as much as environment, although for IPH and DFL, nonlinear selection was significant for hybrids, but not for wilds (Table 3). When the traits were analyzed by the combination of competition and cross type, most of the traits had positive *C*′ values in wilds under competition and negative *C*′ values in hybrids under control conditions, with the opposite being true for DFL (Table 3). In general, PHD and SHD showed the greatest magnitude of selection (Table 3).

**Table 3:** Nonlinear selection coefficients for six sunflower traits calculated by competition (control vs. wheat) treatment, by cross type (wild vs. hybrid), and by the combination of competition and cross type

Trait	By competition					By cross type					By competition and cross type								
	Sig int	Control		Wheat		Sig int	Wild		Hybrid		Sig int	Wild				Hybrid			
												Control		Wheat		Control		Wheat	
		*C*′ ± *SE*		*C*′ ± *SE*			*C*′ ± *SE*		*C*′ ± *SE*			*C*′ ± *SE*		*C*′ ± *SE*		*C*′ ± *SE*		*C*′ ± *SE*	
IPH	***	**−0.09**	*0.03*	**0.04**	*0.00*	***	−0.01	*0.04*	**−0.04**	*0.02*	ns	−0.05	*0.06*	**0.07**	*0.02*	**−0.09**	*0.03*	0.02	*0.01*
ILL	^†^	0.03	*0.04*	**0.04**	*0.01*	***	0.02	*0.03*	−0.01	*0.01*	***	−0.04	*0.08*	**0.06**	*0.01*	**−0.04**	*0.01*	**0.01**	*0.00*
DFL	***	**0.09**	*0.05*	**−0.03**	*0.00*	***	−0.01	*0.04*	**0.05**	*0.02*	***	0.04	*0.09*	**−0.04**	*0.00*	**0.11**	*0.05*	**−0.02**	*0.00*
PHD	***	**−0.31**	*0.06*	−0.03	*0.02*	ns	**0.18**	*0.09*	**−0.10**	*0.02*	ns	0.14	*0.16*	**0.29**	*0.08*	**−0.21**	*0.03*	0.01	*0.01*
SHD	***	**−0.46**	*0.16*	**0.07**	*0.02*	ns	0.24	*0.15*	−0.00	*0.01*	***	−0.58	*0.30*	**0.15**	*0.04*	**−0.05**	*0.03*	**0.04**	*0.00*
THD	*	0.06	*0.06*	**0.08**	*0.02*	***	0.04	*0.08*	0.03	*0.01*	**	−0.10	*0.20*	**0.10**	*0.03*	0.02	*0.03*	**0.03**	*0.00*
		*γ*′ ± *SE*		*γ*′ ± *SE*			*γ*′ ± *SE*		*γ*′ ± *SE*			*γ*′ ± *SE*		*γ*′ ± *SE*		*γ*′ ± *SE*		*γ*′ ± *SE*	
IPH	ns	**−0.09**	*0.04*	−0.05	*0.03*	ns	−0.07	*0.06*	**−0.09**	*0.03*	ns	0.02	*0.08*	−0.06	*0.06*	**−0.15**	*0.04*	−0.01	*0.02*
ILL	ns	0.05	*0.05*	0.00	*0.02*	^†^	0.07	*0.06*	0.00	*0.03*	ns	0.05	*0.09*	0.02	*0.05*	0.00	*0.05*	0.00	*0.02*
DFL	ns	**0.12**	*0.04*	0.03	*0.03*	ns	0.02	*0.05*	0.05	*0.03*	ns	0.09	*0.08*	0.02	*0.05*	0.06	*0.05*	0.05	*0.03*
PHD	ns	**−0.16**	*0.06*	**−0.06**	*0.03*	ns	−0.04	*0.08*	**−0.13**	*0.02*	ns	−0.02	*0.14*	−0.04	*0.06*	**−0.21**	*0.03*	**−0.04**	*0.02*
SHD	***	**−0.55**	*0.13*	0.02	*0.03*	***	**−0.44**	*0.15*	0.00	*0.03*	***	**−0.96**	*0.32*	0.06	*0.06*	0.04	*0.02*	**−0.05**	*0.02*
THD	***	**0.24**	*0.09*	**0.06**	*0.01*	***	**0.32**	*0.09*	0.03	*0.01*	***	0.27	*0.21*	**0.09**	*0.03*	0.02	*0.03*	**0.03**	*0.01*

Note

Standardized nonlinear selection differentials and nonlinear selection gradients with their standard errors (*C' ± SE* and* γ' ± SE,* respectively) are shown. The columns on the left of each section indicate the significance of the relevant interaction (e.g., the stars to the left of the “By competition” section refer to significant interactions between the trait of interest and competition).

We have left BRN out of the results because quadratic coefficient (*C*′) was not significant. Numbers in bold indicate significant coefficients (*p* < 0.05).

Abbreviations: DFL, days to flowering; ILL, intermediate leaf length; IPH, intermediate plant height; ns, nonsignificant; PHD, primary head diameter; SHD, secondary head diameter; THD, tertiary head diameter.

SE values are shown in italic.

^†^ *p* < 0.10.

* *p* < 0.05.

** *p* < 0.01.

*** *p* < 0.001.

With regard to quadratic selection gradients (*γ*′), five out of the six traits (all except ILL) appear to experience significant direct nonlinear selection (Table S3). Specifically, IPH and PHD had negative *γ*′ values and DFL had a positive *γ*′ (Table S3), suggesting the possibility of direct stabilizing and disruptive selection, respectively. In analyses by competition, cross type, and their combination, only SHD and THD showed significant interactions (Table S3). SHD had large negative *γ*′ values under control conditions, while THD had positive *γ*′ values, especially in wilds and under control conditions (Table 3).

## 4 DISCUSSION

Here, we evaluated the phenotypic variation in wild and crop–wild sunflower hybrids under two contrasting environments, as well as dynamics of selection operating on them. While competition and hybridization both affected traits of interest, it was the two‐ and three‐way interactions affecting traits that were salient. For instance, the effect of competition was generally greater for wilds than hybrids, but the nature of the interaction between competition and cross type varied also with the maternal origin of the wild population. Thus, differences between crop and wild plant phenotypes in traits such as intermediate plant height, days to flowering, and number of branches might vary among different crop–wild hybrid zones depending on the competitive environment and the origin of the wild population.

We also found significant directional and nonlinear selection on most traits. Wilds tended to have larger directional selection coefficients, but for fewer traits, than hybrids. Also, directional selection was mostly concentrated in two or three traits without competition, while under competition directional selection was more generalized, but weaker. Number of branches and tertiary head diameter experienced the strongest positive directional selection, being subject to indirect and direct selection in both cross types and environments. When considering nonlinear selection, our data suggest that disruptive selection may be operating under competition, while stabilizing selection may be more common under noncompetitive conditions. For instance, under control conditions, intermediate plant height and secondary head diameter showed negative quadratic selection differentials, suggesting stabilizing selection; while under competitive conditions, these traits reverted to positive quadratic selection differentials, suggestive of disruptive selection. Hence, disruptive selection on some traits may contribute to the origin and maintenance of diversity under competitive conditions (Martin & Pfennig, 2012). Thus, the environmental and genetic context specificity of phenotypes (Mercer et al., 2006) may have translated into context specificity of selection dynamics. The trait‐by‐trait differences in selection dynamics also argue for trait‐specific likelihoods of introgression of crop traits into wild populations.

Our selection analyses accounted for the importance of correlation between traits which can increase, decrease, or even override the selection (magnitude) on a particular trait (Lande & Arnold, 1983; Price & Langen, 1992). For example, the leaf length showed net directional selection and net stabilizing selection associated with both cross types and environments. However, when we looked for direct selection, it was only associated with wheat competition, and quadratic selection was not significant. It is possible that selection in favor of later flowering may select also for larger plant size (Cantamutto et al., 2010; Colautti & Barrett, 2010), thus indirectly favoring traits such as plant height and leaf size. Branching is another trait that showed positive direct selection and even stronger net directional selection indicating that the correlation with other traits increases the apparent selection. For example, later flowering or reduced primary head diameter may play a role in increasing positive selection of branching. By understanding these interactive effects among functional traits on the expression of a higher‐level fitness component (i.e., seed production), we can see the importance of indirect selection (Geber & Griffen, 2003).

### 4.1 Effects of competition, hybridization, and origin on functional traits

When we analyzed our data by origin, we consistently found main effects of competition and hybridization acting on all populations and traits. Yet for very few populations and traits did we find significant G × E interactions between competition and hybridization. The exception would be branching (see Figure 1). Competition reduced branch number, but to a greater degree in wilds than hybrids since wilds produced such high numbers of branches without competition. The plasticity of branching derives from modulation of the activity of axillary meristems after their initiation. These axillary buds can produce dormant buds whose development into branches depends on a complex interplay of external (i.e., competition) and internal (i.e., hormones, developmental stage) factors (McSteen & Leyser, 2005; Teichmann & Muhr, 2015). Wild sunflower has dominant branching (Hockett & Knowles, 1970; Schneiter, Fick, & Miller, 1997). However, the wheat competition in this study may have limited the bud development (especially basal buds), increasing resources allocated to the main shoot and thereby plant height (Teichmann & Muhr, 2015). Thus, most of the variation observed between competition treatments is likely due to plasticity of the trait. By contrast, hybrids had fewer branches even when resources were plentiful; they may have been limited by apical dominance inherited from their crop parent, as seen elsewhere (Snow et al., 1998). In *Erysimun strictum*, simulated competition (with and without soil nutrition) also reduced branching due to increased apical dominance, despite positive directional selection on branch number in all growing conditions (Rautio et al., 2005). Thus, other plants also appear to be meristem‐limited.

Some functional traits in this study expressed a lack of differential effects of competition across cross types within an origin (i.e., there was no G × E interaction). In those cases, we can interpret this as a parallel response of cross types to ecological change. In general, hybrids and wilds both got taller, ended up with shorter leaves, delayed flowering, and reduced secondary head size with competition. Yet for certain populations, we found interesting exceptions that point to cases of genetic variation for traits affected by hybridization that may matter for responses to plant–plant interactions, perhaps indicating variation for adaptation to competitive environments or the surfacing of phenotypic or genetic trade‐offs. For instance, wilds from three origins (ID, WYI, and WYII) delayed flowering to a much greater degree than their hybrid counterparts did when faced with competition. It is interesting to note that these three wild populations were also the ones that flowered earliest under control conditions too, which could have affected their competitive ability due to the resources required for flowering. Similarly, competition greatly reduced the size of hybrid secondary heads, which had a size intermediate between crop and wild heads without competition, such that hybrid heads were often indistinguishable in size from wild ones with competition. Yet, in some populations wild head size was not affected at all by competition. Thus, this G x E interaction affected seed production (Mercer et al., 2006) and the relative fitness of hybrids (Mercer et al., 2007). In recent work, we found that intraspecific competition was especially effective at differentially influencing the relative fitness of hybrids compared to their wild counterparts and chances of crop allele introgression (Mercer et al., 2014).

### 4.2 The role of competition in enhancing selection

Natural selection intensities are known not only to vary over time, but also to be affected by ecological interactions with biotic and abiotic factors. In other words, natural selection can be environmentally dependent. In *Arabidopsis thaliana*, water stress and interspecific competition produced stronger directional selection on early bolting than nonstressful conditions; these ecological stresses also produced some degree of disruptive selection (Brachi, Aimé, Glorieux, Cuguen, & Roux, 2012). Similarly, in *Primula secundiflora,* the nature of selection on flowering time was modified by ecological interactions. Specifically, flowering time was subject to stabilizing selection when the pollinators were syrphid flies, but to disruptive selection when the pollinators were legitimate and illegitimate bumblebees (Wu & Li, 2017).

Competition is an interesting biotic factor to consider with regard to shifting selection pressures since plant‐to‐plant competition, and the manipulation thereof has been such an important force in agricultural change for thousands of years (Ghersa, Roush, Radosevich, & Cordray, 1994). Since the green revolution, agroecosystem management has relied heavily on high crop densities, adequate fertilizer and water, and chemical weed control (Martínez‐Ghersa, Ghersa, & Satorre, 2000; Richards, 2000), and crops have been bred to require these inputs. Thus, such conditions may favor crop‐like phenotypes as compared to wild‐like ones that do not take advantage of those conditions to the same degree. If so, more “agricultural” conditions could hasten introgression of crop‐like traits into wild populations, including adjacent to crop fields. For instance, crop–wild hybrids that establish in agroecosystems may present an admixture of wild and crop traits retaining a high proportion of crop alleles (Casquero, Presotto, & Cantamutto, 2013; Muller et al., 2009; Muller, Latreille, & Tollon, 2011; Presotto et al., 2017). On the other hand, under nonagricultural conditions, wild‐like traits have been shown to be clearly advantageous (Baack et al., 2008; Dechaine et al., 2009) indicating that crop introgression would be lower without than with crop competition. In this sense, a recent study, Corbi, Baack, Dechaine, Seiler, and Burke (2018), found that, under two years of selection in nonagricultural conditions, crop–wild populations evolved to be genetically and phenotypically more wild‐like, suggesting that many crop‐derived traits may be maladapted to wild conditions. That said, there were crop alleles that increased in some regions of the genome. Although stochasticity, especially in small populations, probably contributes to such unpredictable introgression of crop alleles, it is also possible that some crop traits, such as fast early season growth, can be adaptive under most conditions (Mercer et al., 2014, 2007).

In our study, the competitive environment affected whether more crop‐like or wild‐like values of traits were being selected for. Under control conditions, we observed selection (*β'*) toward some wild‐like values of traits for both wilds and hybrids, which could enhance the potential to hasten the regaining of a wild phenotype in a hybridizing population. Specifically, selection was for more branching, later flowering, larger tertiary heads, and smaller primary heads. Plentiful branching and smaller primary heads are more typical of wilds, while later flowering is typical of many wild populations, but not all. Since tertiary heads are absent in the crop and largely absent in F1 hybrids, their presence and size can also be considered more of a wild trait. These strong associations between fitness and wild‐like traits may also account in part for low relative fitness of early‐generation crop–wild hybrids compared to their wild sunflower (Corbi et al., 2018; Mercer et al., 2006, 2007) and radish (Campbell & Snow, 2007) counterparts under wild conditions.

If we look more closely at selection under competition, we observed direct selection (*β'*) on a number of traits, including later flowering, longer leaves, greater branching, and greater secondary and tertiary head diameter, though the strength of all coefficients was less in this environment. While some of the selection operating with competition can be interpreted as being toward crop‐like phenotypes [e.g., longer leaves indicative of faster growth (Mercer, Alexander, & Snow, 2011; Mercer et al., 2007)], selection on later flowering and greater branching would move a population toward wild‐like phenotypes, as we saw in the control conditions. Nevertheless, the reduced strength of selection under competition may potentially lead to a greater possibility for crop allele introgression. This result corroborates results showing the increased relative fitness of hybrids under competition (Mercer et al., 2006).

### 4.3 Selection acting on particular domestication traits

The domestication of sunflower from its wild progenitor, and more recent modern improvement, has led to rapid and dramatic morphological changes in this species. This intense selection fixed several novel quantitative trait phenotypes, for which QTLs have been shown to differ between wild and domesticated types, that is for days to flowering, plant height, branching, achene size, stem diameter, seed dormancy, and self‐compatibility (Burke, Knapp, & Rieseberg, 2005; Burke, Tang, Knapp, & Rieseberg, 2002). These changes have often been useful as morphological markers of crop introgression into wild populations (Cantamutto et al., 2010; Heiser, 1978). These same domestication traits were clear targets of natural selection in our study, but selection differed among traits.

Cultivated sunflowers are well‐known for their large and showy primary heads, whereas wild sunflowers have smaller primary heads. In our work, under control conditions, primary head diameter experienced strong direct selection (*β'*) toward smaller primary heads in wilds. Perhaps, when resources are plentiful, larger primary heads in wild plants may exert stronger apical dominance, thereby reducing the number of secondary and tertiary heads produced on branches (Phillips, 1975). If so, selection may be acting to minimize this. By contrast, under wheat competition, it was only the combination of direct and indirect selection (*s'*) that increased primary head size in both cross types. In fact, hybrids under control conditions appear to be experiencing some form of indirect selection for smaller primary head size, perhaps because those with small primary heads and less apical dominance managed to produce more seeds on branches. This difference in *s'* and *β'* values suggests that there are fewer fitness advantages of greater primary head size under some contexts, but also that accounting for the relationships among traits (e.g., between primary head diameter and higher branching or tertiary head diameter) was important for elucidating selection on this trait. Interestingly, this trait also experienced net stabilizing selection under control conditions (both cross types together), meaning that extreme phenotypes may not always be favored overall. It may be that there are ways that large heads may be associated with apical dominance and the reduction of head number (Phillips, 1975), while small primary heads may be associated with small plants, with neither strategy maximizing fitness. In addition, previous studies have shown that larger heads, which produce large fruits, experience increased pre‐ or post‐dispersal seed predation (Alexander et al., 2001; Dechaine, Burger, & Burke, 2010; Presotto et al., 2016), which may hasten the negative selection under wild conditions, but may also constrain the positive selection under wheat competition.

Flowering time is another trait that has been influenced by domestication, but which also has ecological relevance for natural populations. Crop sunflowers tend to flower early compared to many wild populations, and their flowering period is narrower owing to their single heads. In general, time to flowering is critical in plants and it can be regulated by environmental and genetic factors that accelerate or delay flowering (Takeno, 2016). It appears from our work and others that the environment can also affect the nature of selection on flowering time (Ashworth, Walsh, Flower, Vila‐Aiub, & Powles, 2016; Austen & Weis, 2015; Brachi et al., 2012; Wu & Li, 2017). Under our experimental conditions, direct directional selection (*β' *= 0.08) slightly favored later flowering (a wild‐like trait), under wheat competition. Furthermore, our quadratic selection differentials (*C*′) indicated that plants may have experienced some disruptive selection for flowering time under control conditions, but perhaps some stabilizing selection under wheat competition. From our experience, under competition, early flowering plants could have been negatively affected by resource competition. Yet it is unclear what effect competitive conditions would have for limiting later flowering individuals. Perhaps, later flowering can be problematic due to the universal plant issue of limitations imposed by the arrival of low temperatures late in the season. Thus, stabilizing selection could favor intermediate flowering phenotypes with competition. Without competition, by contrast, disruptive selection for the hybrids would mean that the earliest flowering individuals and the latest ones would do best. It is interesting that we did not find that wilds show this same pattern without competition since two of the latest flowering (Iowa) and earliest flowering (Wyoming II) populations were also the ones that produced the most seed (Mercer et al., 2006). These results may suggest that even when extreme phenotypes are favored, correlated traits are pulling in favor to average flowering time, constraining the evolution of extreme flowering phenotypes under agricultural conditions (Baack et al., 2008; Lande & Arnold, 1983).

The degree and nature of branching strongly determine plant architecture. Branching has also been heavily influenced by domestication in sunflower and other species. While branching is a complex trait governed by multiple loci, environmental variation plays an important role in determining sunflower branching architecture, in particular (Burke, Tang, et al., 2002; Hockett & Knowles, 1970; Nambeesan et al., 2015). In our study, we found that greater branching was strongly directionally selected for in both environments and cross types, indicating that branching plays a major role in fitness. However, our results also indicate lack of nonlinear selection operating on this trait. Thus, it is likely that selection acting over time in a crop–wild hybrid zone would result in advanced generations that resemble wilds in their branching architecture—especially as hybrids backcross with their wild counterparts (Kost et al., 2015).

In summary, our results contribute to our understanding of the dynamics of introgression of crop traits in a number of ways. First, we found that crop competition reduced differences between wild and hybrid cross types for many of the functional traits, such as branching. Since these traits influence fitness, they likely contribute to the increase in relative fitness of crop–wild hybrids compared to wilds under competition seen elsewhere (Mercer et al., 2014, 2006). Second, though wild‐like traits can be selected for in both competitive and noncompetitive environments, we found that more crop‐like traits (e.g., growth traits) were selected for only under competition indicating the possibility of environmental dependence of introgression of crop traits. Third, competitive conditions reduced the magnitude of selection on wild‐like traits (e.g., number of branches and tertiary head diameter)—another indication that crop allele introgression might proceed more easily under such conditions. Other studies have also found that greater competition can accentuate the benefit of crop traits such as seedling size, leaf size, or rapid growth (Campbell et al., 2009; Kost et al., 2015; Mercer et al., 2011, 2014; Owart et al., 2014). Thus, this context‐dependent selection may result in introgression into wild population of crop alleles underlying some traits, but not others.

## CONFLICT OF INTEREST

None declared.

## Data Availability

The data that support the findings of this study are available from the corresponding author upon reasonable request.
