# Supplementary material for: Phenotypic selection under two contrasting environments in wild sunflower and its crop–wild hybrid
Source: Evol Appl. 2019 Jul 1;12(8):1703–17. doi: 10.1111/eva.12828 (PMC6708420; doi:10.1111/eva.12828)
Supplement: Supplementary file 1 [file EVA-12-1703-s001.docx]

Table S1: Traits measured during the experiment at the St. Paul Experiment Station of the University of Minnesota. * trait used as a dependent variable in the selection analyses.

| Traits | Type | Abbreviation | Description |
| --- | --- | --- | --- |
| Early plant height | Metric | EPH | Plant height after 2 weeks of planting. |
|  |  |  |  |
| Intermediate plant height | Metric | IPH | Plant height after 5 weeks of planting. |
|  |  |  |  |
| Early leaf length | Metric | ELL | Leaf length after 2 weeks of planting. |
|  |  |  |  |
| Intermediate leaf length | Metric | ILL | Leaf length after 5 weeks of planting. |
|  |  |  |  |
| Days to flowering | Metric | DFl | Number of days from emergence to flowering (primary head). |
|  |  |  |  |
| Number of Branches | Metric | BRN | Number of branches on the main stem. |
|  |  |  |  |
| Primary head diameter | Metric | PHD | Head diameter of the inflorescence on the top of the stem. |
|  |  |  |  |
| Secundary head diameter | Metric | SHD | Head diameter of the inflorescence on the top of a primary branch. |
|  |  |  |  |
| Tertiary head diameter | Metric | THD | Head diameter of an inflorescence on a secondary branch. |
|  |  |  |  |
| Normal head number | Metric | NHN | Number of normal heads: sum of primary, secondary and tertiary heads. |
|  |  |  |  |
| Deformed head number | Metric | DHN | Number of anomalous heads (fasciation). Related to hybrids. |
|  |  |  |  |
| Appearance | Category | APR | Looks like 0=crop; 1=hybrid poor branched; 2= intermediate hybrid; 3=hybrid well branched; 4=wild. |
| Seeds per reproductive  plant | Metric | * | The sum of the estimated number of seeds produced by all pollinated primary, secondary, tertiary, and deformed heads of the plant. |
|  |  |  |  |
|  |  |  |  |

Table S2: Overall linear selection coefficients with their interactions reflecting phenotypic selections patterns on seven selected traits. Standardized selection differentials and selection gradients with their standard errors (*s' ± SE* and *β' ± SE,* respectively) are shown. ILL: intermediate leaf length; IPH: intermediate plant height, DFL: days to flowering; BRN: number of branches; PHD: primary head diameter; SHD: secondary head diameter; THD: tertiary head diameter. C: competition, CT: cross type; ns: non-significant; *P<0.05; **P<0.01; ***P< 0.001.

| Selection coefficients | **IPH** | | **ILL** | | **DFL** | | **BRN** | | **PHD** | | **SHD** | | **THD** | |
| --- | --- | --- | --- | --- | --- | --- | --- | --- | --- | --- | --- | --- | --- | --- |
|  |  |  |  |  |  |  |  |  |  |  |  |  |  |  |
| ***s´ ± SE*** | 0.04 | *0.02* | 0.18 | *0.02* | 0.03 | *0.02* | 0.60 | *0.02* | -0.11 | *0.03* | 0.14 | *0.03* | 0.55 | *0.02* |
| Source | F-value | Sig. | F-value | Sig. | F-value | Sig. | F-value | Sig. | F-value | Sig. | F-value | Sig. | F-value | Sig. |
| Trait | 6.13 | * | 106.99 | *** | 4.53 | * | 911.53 | *** | 17.26 | *** | 22.14 | *** | 512.62 | *** |
| x CT | 26.99 | *** | 82.27 | *** | 0.62 | ns | 106.04 | *** | 4.60 | * | 3.33 | ns | 172.38 | *** |
| x C | 102.47 | *** | 16.08 | *** | 21.85 | ** | 111.72 | *** | 357.66 | *** | 133.62 | *** | 65.5 | *** |
| x CT x C | 44.47 | *** | 0.22 | ns | 2.35 | ns | 34.13 | *** | 92.77 | *** | 78.70 | *** | 8.72 | ** |
|  |  |  |  |  |  |  |  |  |  |  |  |  |  |  |
| ***β´ ± SE*** | 0.02 | *0.02* | 0.02 | *0.02* | 0.09 | *0.02* | 0.41 | *0.02* | -0.12 | *0.03* | 0.03 | *0.04* | 0.49 | *0.02* |
| Source | F-value | Sig. | F-value | Sig. | F-value | Sig. | F-value | Sig. | F-value | Sig. | F-value | Sig. | F-value | Sig. |
| Trait | 0.72 | ns | 1.08 | ns | 19.01 | *** | 318.00 | *** | 20.01 | *** | 0.78 | ns | 404.52 | *** |
| x CT | 8.45 | ** | 0.28 | ns | 3.02 | ns | 7.99 | ** | 19.18 | *** | 2.49 | ns | 111.32 | *** |
| x C | 4.25 | * | 0.11 | ns | 0.33 | ns | 56.49 | *** | 24.40 | *** | 9.87 | ** | 102.45 | *** |
| x CT x C | 11.01 | *** | 6.04 | * | 2.49 | ns | 10.60 | ** | 20.77 | *** | 0.70 | ns | 28.93 | *** |

Table S3: Overall nonlinear selection coefficients with their interactions reflecting phenotypic selections patterns on seven selected traits. Standardized selection differentials and selection gradients with their standard errors (*C' ± SE* and *γi' ± SE,* respectively) are shown. ILL: intermediate leaf length; IPH: intermediate plant height, DFL: days to flowering; BRN: number of branches; PHD: primary head diameter; SHD: secondary head diameter; THD: tertiary head diameter. C: competition, CT: cross type; ns: non-significant; *P<0.05; **P<0.01; ***P< 0.001.

| Selection coefficients | **IPH** | | **ILL** | | **DFL** | | **BRN** | | **PHD** | | **SHD** | | **THD** | |
| --- | --- | --- | --- | --- | --- | --- | --- | --- | --- | --- | --- | --- | --- | --- |
|  |  |  |  |  |  |  |  |  |  |  |  |  |  |  |
| *C'* ***± SE*** | -0.09 | *0.01* | 0.08 | *0.01* | 0.08 | *0.02* | 0.02 | *0.02* | -0.13 | *0.03* | -0.14 | *0.06* | 0.21 | *0.04* |
| Source | F-value | Sig. | F-value | Sig. | F-value | Sig. | F-value | Sig. | F-value | Sig. | F-value | Sig. | F-value | Sig. |
| Trait | 38.63 | *** | 24.46 | *** | 17.67 | *** | 0.08 | ns | 15.92 | *** | 4.61 | * | 26.89 | *** |
| x CT | 21.67 | *** | 41.10 | *** | 25.50 | *** | 0.72 | ns | 0.00 | ns | 3.41 | ns | 28.18 | *** |
| x C | 54.81 | *** | 3.30 | ns | 37.23 | *** | 0.01 | ns | 20.10 | *** | 23.70 | *** | 6.35 | * |
| x CT x C | 3.72 | ns | 13.36 | *** | 38.33 | *** | 2.60 | ns | 0.15 | ns | 14.06 | *** | 12.97 | *** |
|  |  |  |  |  |  |  |  |  |  |  |  |  |  |  |
| *γi'****± SE*** | -0.07 | *0.03* | 0.05 | 0.03 | 0.07 | *0.03* | - | *-* | -0.18 | *0.03* | -0.29 | *0.06* | 0.22 | *0.04* |
| Source | F-value | Sig. | F-value | Sig. | F-value | Sig. | F-value | Sig. | F-value | Sig. | F-value | Sig. | F-value | Sig. |
| Trait | 4.63 | * | 2.51 | ns | 5.88 | * | - | - | 28.44 | *** | 20.71 | *** | 34.39 | *** |
| x CT | 1.74 | ns | 3.27 | ns | 2.56 | ns | - | - | 0.75 | ns | 19.11 | *** | 28.40 | *** |
| x C | 0.49 | ns | 2.12 | ns | 3.05 | ns | - | - | 1.56 | ns | 22.87 | *** | 13.73 | *** |
| x CT x C | 0.55 | ns | 2.58 | ns | 2.86 | ns | - | - | 1.17 | ns | 30.65 | *** | 13.82 | *** |
